# Drug-induced hearing disorders: a disproportionality analysis of the FAERS database

**DOI:** 10.3389/fphar.2024.1480994

**Published:** 2024-11-22

**Authors:** Baojian Li, Xiaoling Hu, Zichen Yue

**Affiliations:** Department of Pharmacy, Heping Hospital Affiliated to Changzhi Medical College, Changzhi, China

**Keywords:** hearing disorders, adverse event, FAERS, disproportionality analysis, signal mining

## Abstract

**Background:**

To evaluate and identify reports of adverse events related to hearing impairment with drugs approved in the past 20 years, to identify new adverse reaction signals related to hearing impairment that have not yet been reported, and to improve the safety of drug treatments.

**Methods:**

The adverse event report data from the FAERS database from the first quarter of 2004 to the fourth quarter of 2023 were retrieved. “Hearing disorders” was used as the keyword to screen for drugs related to the adverse event. After standardizing the drug name and the adverse drug event name, the adverse event reports with hearing disorders as the main suspect were collected, and the proportional imbalance algorithm was used to detect the potential adverse event signals to drug-related hearing impairment.

**Results:**

The top five drugs with the highest number of reported adverse events to hearing impairment were sacubitril/valsartan (2,674), adalimumab (2,479), etanercept (1,834), tofacitinib (1,812), and apixaban (1,600). Except for adalimumab, the risk of hearing impairment is not mentioned in the instructions. The top five drugs for new signal strength are pancuronium (*n* = 13, ROR 67.57, PRR 53.61, IC5.74, EBGM 53.06), paromomycin (*n* = 6, ROR 46.3, PRR 39.33, IC5.30, EBGM 39.33), tafamidis (*n* = 300, ROR 14.90, PRR 14.13, IC3.82, EBGM 14.07), vildagliptin/metformin (*n* = 83, ROR 11.47, PRR 11.02, IC3.46, EBGM 11.01), and atorvastatin calcium/ezetimibe (*n* = 6, ROR 10.76, PRR 10.36, IC3.37, EBGM 10.36).

**Conclusion:**

Our study covered 20 years of real-world data on reports of adverse events related to hearing impairment in the FAERS database, validating previous reports and studies, as well as identifying drugs that signal new adverse events of hearing impairment, especially some drugs commonly used for the treatment of chronic diseases (a combination of hypoglycemic drugs, antihypertensive drugs, and lipid modulators) and some new drugs in the 5-year post-market period.

## Introduction

Drug-induced ototoxicity has significant public health implications. Despite the evidence, many healthcare professionals may be unaware of the ototoxicity risks of many drugs, especially those that have been on the market for less than 5 years. Drug-induced ototoxicity is a dysfunction of the inner ear (cochlea and/or vestibular organ) or the eighth cranial nerve or is associated with oxidative phosphorylation-related genes and integrin-related genes (Tanaka et al., 2021) and may also be associated with mitochondrial dysfunction and consequent oxidative stress in cochlear cells (Tan and Song, 2023). Drug-induced ototoxicity may be underestimated for a long time, often until irreversible hearing loss develops, and platinum derivatives and aminoglycosides are particularly associated with permanent hearing loss. The most commonly prescribed drugs that cause ototoxicity are anticancer drugs, aminoglycoside antimicrobials, cyclic diuretics, calcium channel blockers, nonsteroidal anti-inflammatory drugs, antimalarials, and salicylic acids (Wang Y. et al., 2024), as well as antimalarials, antineoplastic drugs, and some topical drugs (Seligmann et al., 1996). At present, the signal pooling studies of adverse events related to hearing impairment are either 5 years old (Altissimi et al., 2020; Favrelière et al., 2020) or studies of individual drugs, and there is a lack of signal mining studies on the reports of drug-induced adverse events related to hearing impairment in the past 5 years. The aim of our study was to use the United States Food and Drug Administration’s Adverse Event Reporting System (FAERS) database to evaluate reports of adverse events related to hearing impairment related to drugs approved in the past 20 years, to identify new signals of adverse events associated with hearing impairment that have not yet been reported, and to improve the safety of drug treatments.

## Materials and methods

### Data sources and procedures

Adverse event data for this study were obtained from the FAERS database (https://fis.fda.gov/extensions/FPD-QDE-FAERS/FPD-QDE-FAERS.html). The database has been publicly available since 2004, and this study downloaded adverse event reporting data from the United States Standard Code for Information Interchange (ASCII) FAERS database from the first quarter of 2004 to the fourth quarter of 2023.

For data downloaded from the FAERS database with the same “caseid” (report code), only the most recent report based on the date is retained, and duplicate reports are removed. The drug name is standardized according to the RxNorm drug standardized naming system to standardize the drug name in the FAERS data. The International Dictionary of Medical Terms version 26.1 (MedDRA 26.1) was used to match the primary terms (PT) for adverse events of hearing impairment. The word “hearing disability” was used as a keyword to screen for drug names associated with this adverse event and aggregate various “hearing disorders” (including ototoxicity, auditory disorder, diplacusis, dysacusis, paracusis, and conductive) deafness, deafness, deafness bilateral, deafness neurosensory, deafness permanent, deafness transitory, deafness unilateral, hypoacusis, mixed deafness, neurosensory hypoacusis, sudden hearing loss, and audiogram abnormal.

After standardizing the drug name and the name of the adverse event, adverse event reports with hearing disorders and a primary suspect (PS) drug were collected. These adverse event reports were characterized by gender, age, indication diagnosis, time of occurrence, reporting person, reporting country, and outcome.

### Signal analysis algorithms

In this article, a disproportional analysis (DPA) algorithm commonly used in pharmacovigilance studies was used to detect the potential signals of adverse events of hearing impairment with related drugs. The proportional imbalance algorithm is a widely used data mining method that analyzes the degree of correlation between drugs and adverse reactions by comparing the frequency ratios observed in exposed and unexposed populations using a four-grid table (Table 1). In this study, the reporting odds ratio (ROR), the proportional reporting ratio (PRR), the Bayesian confidence propagation neural network (BCPNN), and the empirical Bayesian geometric mean (EBGM) were used to calculate the signal strength. The results of adverse reaction signals should meet the positive signal discrimination criteria of the above four algorithms (Table 2) to be judged as valid signals. The effective signal is judged to be a new adverse event signal after checking for an adverse reaction not mentioned in the FDA drug instructions (https://www.accessdata.fda.gov/scripts/cder/daf/index.cfm). All data in this study were processed and statistically analyzed using R4.4.0 and MS Excel software.

**TABLE 1:** Four-fold table of disproportionality measurement.

	Drug-related AEs	Non-drug-related AEs	Total
Drug	a	b	a + b
Non-drug	c	d	c + d
Total	a + c	b + d	N = a + b + c + d

a: number of reports containing both the suspect drug and the suspect adverse event.

b: number of reports containing the suspect adverse event with other medications (except the drug of interest).

c: number of reports containing the suspect drug with other adverse events (except the event of interest).

d: number of reports containing other drugs and other adverse events.

**TABLE 2:** ROR, PRR, BCPNN, and EBGM formulas and thresholds.

Method	Formula	Threshold
ROR	$\text{ROR}=\frac{a\;/\;c}{b\;/\;d}.$	a ≥3 ROR ≥3 95%CI (lower limit) > 1
	$SE\left(lnROR\right)=\sqrt{\frac{1}{a}+\frac{1}{b}+\frac{1}{c}+\frac{1}{d.}}$	
	$95\%CI=e^{\ln\left(ROR\right)\pm 1.96se}.$	
PRR	$\text{PRR}=\frac{a\;\frac{}{\;\left(a+b\right)}.}{c\;/\;\left(c+d\right)}$	a ≥3 PRR ≥2 95%CI (lower limit) > 1
	$SE\left(lnPRR\right)=\sqrt{\frac{1}{a}-\frac{1}{a+b}+\frac{1}{c}-\frac{1}{c+d.}}$	
	$95\%CI=e^{\ln\left(PRR\right)\pm 1.96se}.$	
BCPNN	$\text{IC}=\log_{2}\frac{p\left(x,y\right)}{p\left(x\right)p\left(y\right)}=\log_{2}\frac{a\left(a+b+c+d\right).}{\left(a+b\right)\left(a+c\right)}$ $E\left(\text{IC}\right)=\log_{2}\frac{\left(a+\gamma 11\right)\left(a+b+c+d+\alpha \right)\left(a+b+c+d+\beta \right).}{\left(a+b+c+d+\gamma \right)\left(a+b+\alpha 1\right)\left(a+c+\beta 1\right)}$ $V\left(\text{IC}\right)=\frac{1}{{\left(\ln2\right)}^{2}}\left[\frac{\left(a+b+c+d\right)-a+\gamma -\gamma 11}{\left(a+\gamma 11\right)\left(1+a+b+c+d+\gamma \right)}+\frac{\left(a+b+c+d\right)-\left(a+b\right)+a-\alpha 1}{\left(a+b+\alpha 1\right)\left(1+a+b+c+d+\alpha \right)}\;\gamma \;.=\gamma 11\frac{\left(a+b+c+d+\alpha \right)\left(a+b+c+d+\beta \right)}{\left(a+b+\alpha 1\right)\left(a+c+\beta 1\right)}\right]$	IC025 > 0
	$\text{IC}-2\text{SD}=E\left(\text{IC}\right)-2\;\sqrt{V\left(\text{IC}\right)}.$	
EBGM	$E\text{BGM}=\frac{a\left(a+b+c+d\right).}{\left(a+c\right)\left(a+b\right)}$	EBGM05 > 2
	$SE\left(lnEBGM\right)=\sqrt{\frac{1}{a}+\frac{1}{b}+\frac{1}{c}+\frac{1.}{d}}$	
	$95\%CI=e^{\ln\left(EBGM\right)\pm 1.96se.}$	

## Results

### Basic characteristics of adverse events related to hearing impairment

**FIGURE 1:**
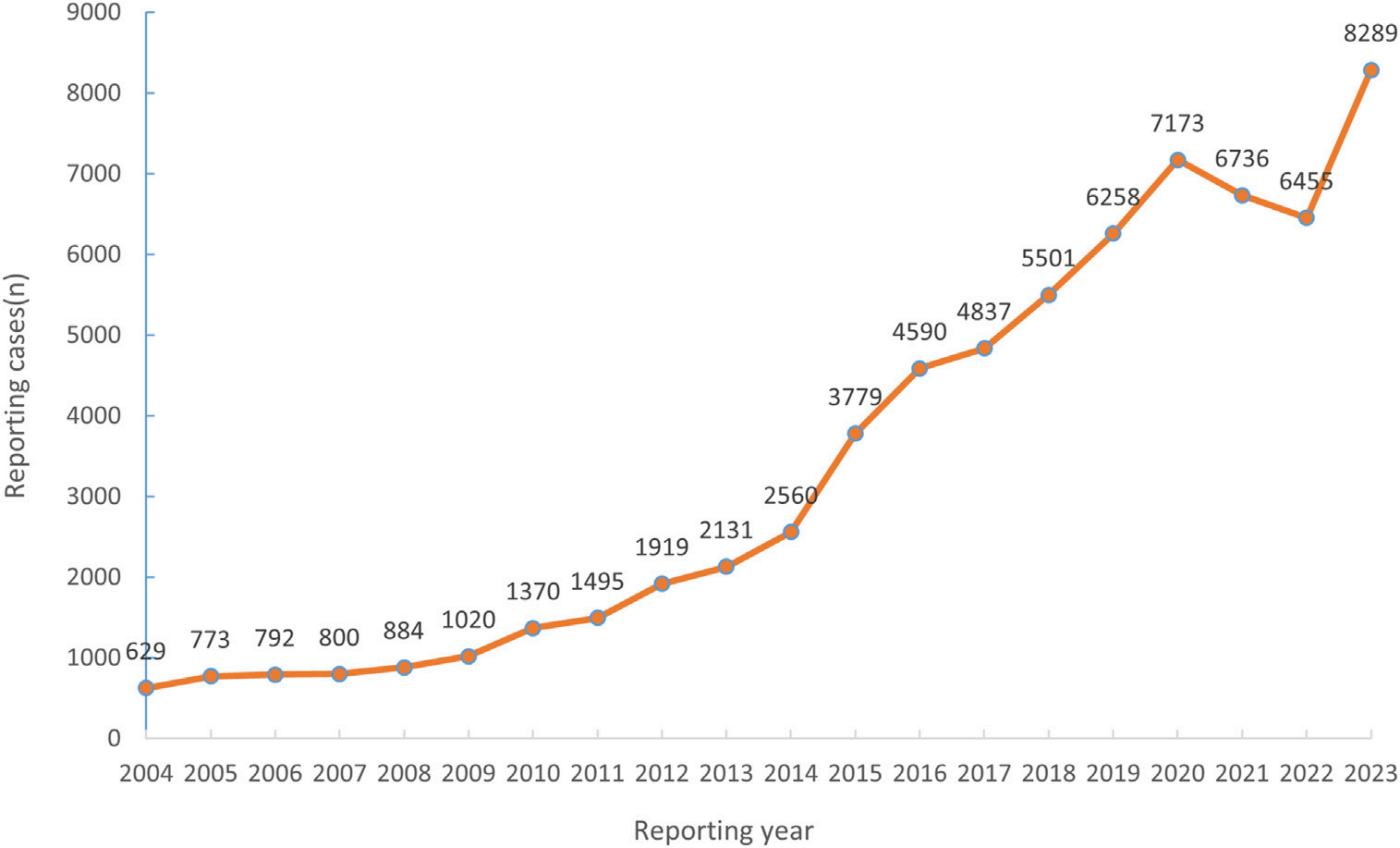
Number of reported cases of drug-induced hearing disorders from Q1 2004 to Q4 2023.

As of the fourth quarter of 2023, the number of reports of adverse events related to hearing impairment in the FAERS database was 95,901, and the number of adverse events reported with hearing impairment as the preferred term was 67,991. From the first quarter of 2004 to the fourth quarter of 2023, the number of reports of adverse events with hearing impairment as the preferred term increased year by year (Figure 1).

Table 3 showed the characteristics of the population with adverse events to hearing impairment, in which 52.99% were females, 40.24% were males, and 6.77% were unknown in gender. People aged 65 years and older accounted for the largest proportion (30.28%) of the adverse events reported by known age. The greatest number of adverse events to hearing impairment occurred within 1 week after medication (6.03%). The top three indications were rheumatoid arthritis (5.58%), multiple sclerosis (3.97%), and type 2 diabetes mellitus (2.47%).

**TABLE 3:** Clinical characteristics of reports with hearing disorders from the FAERS database.

Variable	Reports, n (%)
Sex	
Female	36,029 (52.99)
Male	27,362 (40.24)
Unknown	4,600 (6.77)
Age (y)	
<19	1,668 (2.45)
19–65	15,411 (22.67)
≥65	20,585 (30.28)
Unknown	30,327 (44.60)
Outcomes	
Hospitalization	11,403 (18.30)
Disability	6,054 (9.71)
Death	1,461 (2.34)
Life-threatening	1,132 (1.82)
Congenital anomaly	846 (1.36)
Required intervention to prevent permanent impairment/damage	408 (0.65)
Other serious	41,016 (65.82)
Time of occurrence	
≤ 1 week	4,101 (6.03)
>1 week, ≤1 month	1,340 (1.97)
>1 month, ≤1 year	3,499 (5.15)
>1 year	2,541 (3.74)
Unknown	56,510 (83.11)
Indication	
Rheumatoid arthritis	3,795 (5.58)
Multiple sclerosis	2,698 (3.97)
Type 2 diabetes mellitus	1,678 (2.47)
Others	45,514 (66.94)
Product used for unknown indication	14,306 (21.04)
Reporter	
Consumer	42,274 (62.18)
Physician	11,448 (16.84)
Pharmacist	6,115 (8.99)
Other health professional	5,440 (8.00)
Unknown	2,210 (3.25)
Lawyer	486 (0.71)
Registered nurse	18 (0.03)
Reported countries	
United States	38,784 (57.04)
Canada	2,974 (4.37)
United Kingdom	2,165 (3.18)
Germany	1,360 (2.00)
France	1,333 (1.96)
Japan	1,217 (1.79)
Brazil	1,215 (1.79)
China	427 (0.63)
Netherlands	394 (0.58)
Colombia	389 (0.57)
Other	17,733 (26.08)

**FIGURE 2:**
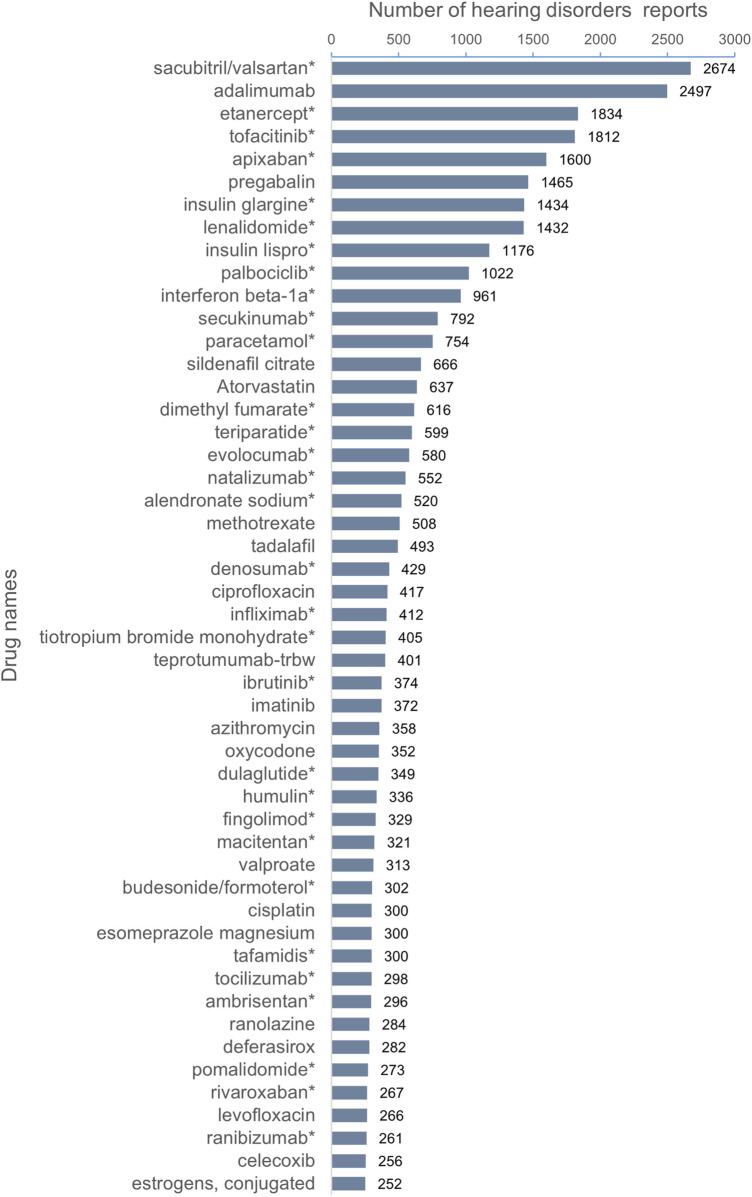
Top 50 drugs with the highest number of reported hearing disorders. ^*^The package insert did not suggest risk for hearing disorders.

The top 50 drugs with the highest number of adverse events to hearing impairment are shown in Figure 2. The top five drugs were sacubitril/valsartan (2,674 reports), adalimumab (2,479 reports), etanercept (1,834 reports), tofacitinib (1,812 reports), and apixaban (1,600 reports). None of these drugs mentioned hearing impairment risk except the adalimumab label. Among the top 50 drugs with the highest number of adverse events to hearing impairment, 31 drug labels did not mention the risk of hearing impairment.

### Signal detection of adverse events related to hearing impairment

The statistical results of disproportionality analysis showed that among the top 35 drugs in the signal strength detection of adverse events, 19 drug labels did not mention the risk of hearing impairment in the drug labels, which was a new adverse event signal. The top five drugs for new signal strength are pancuronium [*n* = 13, ROR 67.57 (36.66, 124.55), PRR 53.61 (32.84, 87.51), IC5.74 (4.91), EBGM 53.06 (32.13)], paromomycin [*n* = 6, ROR 46.3 (19.40, 110.51), PRR 39.33 (18.68, 82.83), IC5.30 (4.14), EBGM 39.33 (18.99)], tafamidis [*n* = 300, ROR 14.90 (13.26, 16.74), PRR 14.13 (12.56, 15.89IC3.82 (3.65), EBGM 14.07 (12.77)], vildagliptin/metformin [*n* = 83, ROR 11.47 (9.21, 14.30), PRR 11.02 (8.88, 13.67), IC3.46 (3.15), EBGM 11.01 (9.16)], and atorvastatin calcium/ezetimibe [*n* = 6, ROR 10.76 (4.75, 24.36), PRR 10.36 (4.73, 22.69), IC3.37 (2.28), EBGM 10.36 (5.23)]. The top five drugs with the highest number of reports and no mention of this adverse event in the label were sacubitril/valsartan [*n* = 2,674, ROR 7.51 (7.22, 7.81), PRR 7.33 (7.05, 7.62), IC2.82 (2.77), EBGM 7.08 (6.85)], tafamidis [*n* = 300, ROR 14.90 (13.26, 16.74), PRR 14.13 (12.56, 15.89), IC3.82 (3.65), EBGM 14.07 (12.77)], pamidronate disodium [*n* = 102, ROR 6.98 (5.73, 8.50), PRR 6.82 (5.61, 8.3), IC2.77 (2.49), EBGM 6.81 (5.78)], dofetilide [*n* = 97, ROR 4.63 (3.79, 5.66), PRR 4.57 (3.76, 5.56), IC2.19 (1.90), EBGM 4.56 (3.86)], and vildagliptin/metformin [*n* = 83, ROR 11.47 (9.21, 14.30), PRR 11.02 (8.88, 13.67), IC3.46 (3.15), EBGM 11.01 (9.16)]. The signal intensity of teprotumumab, ranolazine, amikacin, and gentamicin ranked in the top 35, and the number of reports was >100. However, adverse events related to hearing impairment were indicated in the instructions (Table 4).

**TABLE 4:** Top 35 drugs for signal strength.

Drug name	Case reports	ROR (95% CI)	PRR (95% CI)	Chi sq	IC(IC025)	EBGM(EBGM05)	Package insert suggests risk for hearing disorders
Pancuronium	13	67.57 (36.66, 124.55)	53.61 (32.84, 87.51)	673.71	5.74 (4.91)	53.06 (32.13)	N
Teprotumumab	401	46.49 (41.79, 51.73)	39.05 (35.81, 43.57)	15,019.08	5.30 (5.14)	39.28 (35.92)	Y
Paromomycin	6	46.30 (19.40, 110.51)	39.33 (18.68, 82.83)	225.01	5.30 (4.14)	39.33 (18.99)	N
Tafenoquine	5	31.06 (12.27, 78.59)	27.79 (12.20, 63.30)	129.62	4.80 (3.57)	27.79 (12.78)	Y
Ethacrynic acid	7	27.85 (12.76, 60.78)	25.21 (12.45, 51.05)	163.34	4.66 (3.60)	25.20 (13.12)	Y
Brinzolamide/timolol maleate	12	20.37 (11.32, 36.68)	18.94 (10.94, 32.79)	204.67	4.24 (3.43)	18.94 (11.58)	Y
Tafamidis	300	14.90 (13.26, 16.74)	14.13 (12.56, 15.89)	3659.12	3.82 (3.65)	14.07 (12.77)	N
Neomycin	15	14.58 (8.67, 24.54)	13.85 (8.48, 22.61)	179.44	3.79 (3.06)	13.84 (8.96)	Y
Streptomycin	5	13.99 (5.69, 34.43)	13.32 (5.62, 31.55)	57.17	3.73 (2.55)	13.31 (6.27)	Y
Gentamicin	120	13.61 (11.32, 16.35)	12.97 (10.87, 15.47)	1328.56	3.69 (3.43)	12.95 (11.10)	Y
Amikacin	233	12.81 (11.22, 14.61)	12.24 (10.88, 13.77)	2406.54	3.61 (3.42)	12.2 (10.93)	Y
Vildagliptin/metformin	83	11.47 (9.21, 14.30)	11.02 (8.88, 13.67)	758.50	3.46 (3.15)	11.01 (9.16)	N
Atorvastatin calcium/ezetimibe	6	10.76 (4.75, 24.36)	10.36 (4.73, 22.69)	50.96	3.37 (2.28)	10.36 (5.23)	N
Quinine	32	10.75 (7.55, 15.32)	10.36 (7.42, 14.46)	271.53	3.37 (2.87)	10.36 (7.70)	Y
Calcium polycarbophil	3	8.78 (2.78, 27.76)	8.52 (2.79, 26.04)	19.99	3.09 (1.65)	8.52 (3.25)	N
Ranolazine	284	7.62 (6.77, 8.58)	7.43 (6.61, 8.36)	1580.53	2.89 (2.72)	7.41 (6.71)	Y
Sacubitril/valsartan	2,674	7.51 (7.22, 7.81)	7.33 (7.05, 7.62)	14085.08	2.82 (2.77)	7.08 (6.85)	N
Pamidronate disodium	102	6.98 (5.73, 8.50)	6.82 (5.61, 8.3)	507.69	2.77 (2.49)	6.81 (5.78)	N
Deferoxamine	26	6.90 (4.67, 10.19)	6.74 (4.64, 9.78)	127.63	2.75 (2.20)	6.74 (4.87)	Y
Clofazimine	28	6.23 (4.28, 9.06)	6.10 (4.20, 8.85)	119.93	2.61 (2.08)	6.10 (4.46)	N
Epoetin alfa-epbx	31	6.22 (4.36, 8.89)	6.10 (4.29, 8.68)	132.59	2.61 (2.10)	6.10 (4.52)	N
Ofloxacin	33	6.11 (4.33, 8.63)	5.99 (4.29, 8.36)	137.64	2.58 (2.09)	5.99 (4.48)	Y
Travoprost	97	5.96 (4.87, 7.29)	5.85 (4.81, 7.12)	390.85	2.55 (2.26)	5.84 (4.94)	Y
Bedaquiline	47	5.96 (4.46, 7.96)	5.85 (4.45, 7.70)	189.47	2.55 (2.13)	5.84 (4.59)	N
Filgrastim-aafi	7	5.86 (2.77, 12.41)	5.75 (2.78, 11.87)	27.60	2.52 (1.51)	5.75 (3.07)	N
Capmatinib	43	5.60 (4.14, 7.58)	5.50 (4.10, 7.38)	158.92	2.46 (2.03)	5.50 (4.27)	N
Vinblastine	4	5.42 (2.01, 14.59)	5.33 (2.04, 13.93)	14.11	2.41 (1.13)	5.33 (2.33)	Y
Avanafil	9	4.99 (2.58, 9.66)	4.92 (2.58, 9.39)	28.19	2.30 (1.39)	4.92 (2.83)	N
Cilastatin sodium/imipenem	10	4.98 (2.67, 9.32)	4.91 (2.67, 9.01)	31.23	2.29 (1.43)	4.91 (2.91)	Y
Tepotinib	6	4.79 (2.14, 10.74)	4.72 (2.16, 10.34)	17.66	2.24 (1.16)	4.72 (2.40)	N
Tadalafil	493	4.76 (4.36, 5.21)	4.69 (4.34, 5.07)	1428.64	2.22 (2.09)	4.67 (4.33)	Y
Ranitidine	15	4.72 (2.83, 7.87)	4.65 (2.79, 7.74)	43.20	2.22 (1.50)	4.65 (3.04)	N
Teriflunomide	42	4.67 (3.44, 6.33)	4.60 (3.43, 6.17)	118.74	2.20 (1.77)	4.60 (3.56)	N
Dofetilide	97	4.63 (3.79, 5.66)	4.57 (3.76, 5.56)	270.89	2.19 (1.90)	4.56 (3.86)	N
Glycopyrrolate/indacaterol	25	4.59 (3.09, 6.82)	4.53 (3.06, 6.70)	69.03	2.18 (1.62)	4.53 (3.25)	N

## Discussion

This study was derived from real-world data from the FAERS database about adverse events reported from the first quarter of 2004 to the fourth quarter of 2023. The full text follows the explanation and elaboration of READUS-PV (Fusaroli et al., 2024). We defined the 18 preferred terms in MedDRA 26.1 for adverse events diagnosed as hearing impairment as “hearing impairment” and found that the number of reported adverse events related to hearing impairment increased year by year. Among the top 50 drugs with the highest number of reports, 31 drugs did not mention the risk of hearing impairment, of which the top three reports were sacubitril/valsartan (2,674), etanercept (1,834), and tofacitinib (1,812). Among the top 35 drugs with the potential signal intensity of adverse events to hearing impairment detected by the proportional imbalance detection method, 16 drugs have mentioned the risk of hearing impairment in the instructions, including drugs with known ototoxicity, including aminoglycosides (neomycin, streptomycin, gentamicin, and amikacin), highly effective diuretics (ethacrynic acid), antimalarial drugs (tafenoquine and quinine), antimicrobial drugs (ofloxacin and cilastatin sodium/imipenem), glaucoma treatment drugs (travoprost and brinzolamide/timolol maleate), antineoplastic drugs (vinblastine), phosphodiesterase-5 inhibitor (tadalafil), iron chelator (deferoxamine), anti-angina drug (ranolazine), as well as a new drug teprotumumab released within the last 5 years. Nineteen drugs do not mention the risk of hearing impairment in the drug label, including drugs for the treatment of chronic diseases, such as vildagliptin/metformin, atorvastatin calcium/ezetimibe, sacubitril/valsartan, and glycopyrrolate/indacaterol. Some new drugs in the 5-year post-market period, including tafamidis, epoetin alfa-epbx, capmatinib, and tepotinib, are particularly concerned about the risk of hearing impairment with the use of the medication.

Sacubitril/valsartan contains sacubitril (an enkephalinase inhibitor) and valsartan (an angiotensin receptor antagonist). Sacubitril/valsartan is a drug commonly used for the treatment of high blood pressure. We found that sacubitril/valsartan is the drug with the most reported hearing impairment events and is a new signal. The sacubitril/valsartan combination was first approved and registered in the United States of America (USA) on 7 July 2015. By December 2023, sacubitril/valsartan reported the highest number of adverse events (2,674), which may be related to the implementation of certain policies by pharmaceutical companies. Palffy and Lewis (2024) pointed out that patient support programs (PSPs) are used by the pharmaceutical industry to provide education and support to consumers to overcome the challenges they face in managing their condition and treatment. However, by their nature, PSPs inevitably generate adverse event reports. Wang and Liu (2024) mentioned adverse events of hearing loss may be related to adverse events with labeled diuretics used in the treatment of congestive heart failure. This requires further analysis of the association between drug combinations or drug interactions with hearing impairment. Other chronic disease drugs, such as vildagliptin/metformin, atorvastatin calcium/ezetimibe, and glycopyrrolate/indacaterol, also need to be considered for such related studies. Note that type 1 diabetes is associated with impairment of the auditory system (Mittal et al., 2024), and a higher vascular risk is highly associated with idiopathic sudden sensorineural hearing loss (Simões et al., 2023). Therefore, in this study, there was a protopathic bias in the reporting of adverse events caused by common drugs for chronic diseases, such as sacubitril/valsartan and vildagliptin/metformin. Because hearing loss is associated with aging (Reisinger and Weisz, 2024), our study showed that hearing loss occurred more frequently in people aged 65 years and older, who accounted for the largest proportion (30.28%) of the adverse events reported by known age. Therefore, our study can only investigate associations, not infer causation, and cannot completely rule out the possibility of primary bias.

Tafamidis is the first medicine licensed for treating wild-type and hereditary transthyretin amyloid cardiomyopathy. The hearing impairment caused by tafamidis is a new adverse event signal revealed by our study. Consistent with our study, Li et al. (2024) found that tafamidis’ adverse events for hearing impairment in the FAERS database were significant adverse events that have not been previously documented in clinical trials or drug inserts. Although tafamidis works by reducing amyloid formation, hearing may be affected if the medication is not effective or if amyloid is deposited in the ear structure due to some unknown factor. Epoetin alfa-epbx is a type of erythropoietin (EPO). Animal experiments conducted by Frederiksen et al. (2007) indicated that erythropoietin may augment noise-induced hearing loss. This is contradictory to the beneficial effect of EPO, which has been reported by most studies on stressed neural tissues. EPO administration may alter the blood flow dynamics of the cochlear vascular bed during or after noise exposure by a potential induction of vasoconstriction. This explains the possible causes of epoetin alfa-epbx’s hearing impairment at the animal level. Capmatinib is a potent and selective inhibitor of the mesenchymal–epithelial transition, approved in 2020 for the treatment of metastatic non-small cell lung cancer. Qi et al. (2024) identified new major adverse event signals in capmatinib, one of which was deafness, through data mining of the FDA’s Adverse Event Reporting System database. Li and Wang (2024) revealed a significant correlation between capmatinib use and the incidence of hearing loss, which is consistent with our discovery that hearing impairment is a new signal of capmatinib. Tepotinib is also a potent and selective inhibitor of the mesenchymal–epithelial transition, approved in 2021 by the FDA for the treatment of metastatic non-small cell lung cancer. At present, there have been no reports of adverse events with hearing impairment.

The phosphodiesterase type 5 (PDE-5) inhibitors sildenafil, vardenafil, and tadalafil are mainly used to treat erectile dysfunction, and sildenafil is also indicated for pulmonary hypertension. Wang et al. (2024a) investigated the potential risk of sudden hearing loss with sildenafil. Snodgrass et al. (2010) reported that vardenafil may cause hearing loss. Our study found 666 reports of adverse reactions related to hearing impairment in sildenafil and detected new signals of hearing impairment in avanafil.

Teprotumumab is an inhibition of the insulin-like growth factor 1 receptor (IGF-1R), which reverses thyroid eye disease. Wang et al. (2024b) found that teprotumumab was most reported in the ear and labyrinthine disorders of acoustic emission signals. Other cumulative case reports and series suggest that teprotumumab may significantly increase the risk of hearing impairment (Zhang et al., 2024). We also found that teprotumumab had a high number of adverse reactions to hearing impairment (401) and a high signal intensity (ROR = 46.49).

Tanaka et al. (2019) observed the risk of drug-induced hearing loss with pancuronium bromide in patients with congenital diaphragmatic hernia by using the Japan Adverse Drug Event Reporting (JADER) database to assess the risk of drug-induced hearing loss. Our study found that pancuronium caused adverse events of hearing impairment as a new strong signal. Bedaquiline is particularly used for the treatment of multidrug-resistant tuberculosis. An important finding of Wu et al. (2024) was the identification of adverse events associated with ear and labyrinth disease. Bedaquiline-induced hearing impairment is also a new adverse event signal revealed by our study. Secukinumab is approved for the treatment of psoriasis, psoriatic arthritis, and ankylosing spondylitis. Shu et al. (2022) detected adverse reaction signals such as ear and labyrinth disease, while our study found that 792 reports of adverse events related to hearing impairment were not valid signals after being detected by the proportional imbalance test.

This study also has shortcomings and limitations. First, the study relies on FAERS, a spontaneous reporting system, which itself introduces reporting bias due to incomplete data capture, potential false negatives, and delayed input. Second, it is difficult to establish causation from these data, as adverse event reporting does not equate to confirmed causation. Third, a query based on the Standardized MedDRA Query (SMQ) term “hearing impairment” would have been preferable and more reliable than selecting a few preferred terms. Finally, the data results were not compared and validated in other adverse event databases. These shortcomings require further research. Nonetheless, real-world studies have provided valuable information on the use of drugs associated with adverse events in hearing impairment in a wide range of patient populations that can be used to ensure drug safety.

In conclusion, our study covered 20 years of real-world data on adverse reactions related to hearing impairment reported in the FAERS database, validated previous reports and studies (Tan and Song, 2023; Altissimi et al., 2020; Favrelière et al., 2020; Ostroumova et al., 2019; Rizk et al., 2020; Lanvers-Kaminsky et al., 2017), and identified drugs that signal new adverse events of hearing impairment, especially some drugs commonly used for the treatment of chronic diseases (a combination of hypoglycemic drugs, antihypertensive drugs, and lipid modulators) and some new drugs in the 5-year post-market period. Healthcare providers prescribing drugs with adverse events associated with hearing impairment should monitor patients’ hearing during the treatment. Because the proportional imbalance detection method does not represent a causal relationship, more clinical practice is needed to validate and find ways and interventions to reduce and prevent adverse reactions related to hearing impairment. Overall, our findings provide a warning for healthcare workers to identify and manage drugs associated with adverse events of hearing impairment, ultimately ensuring patient safety during drug treatment.

## Data Availability

The original contributions presented in the study are included in the article/supplementary material; further inquiries can be directed to the corresponding author.
